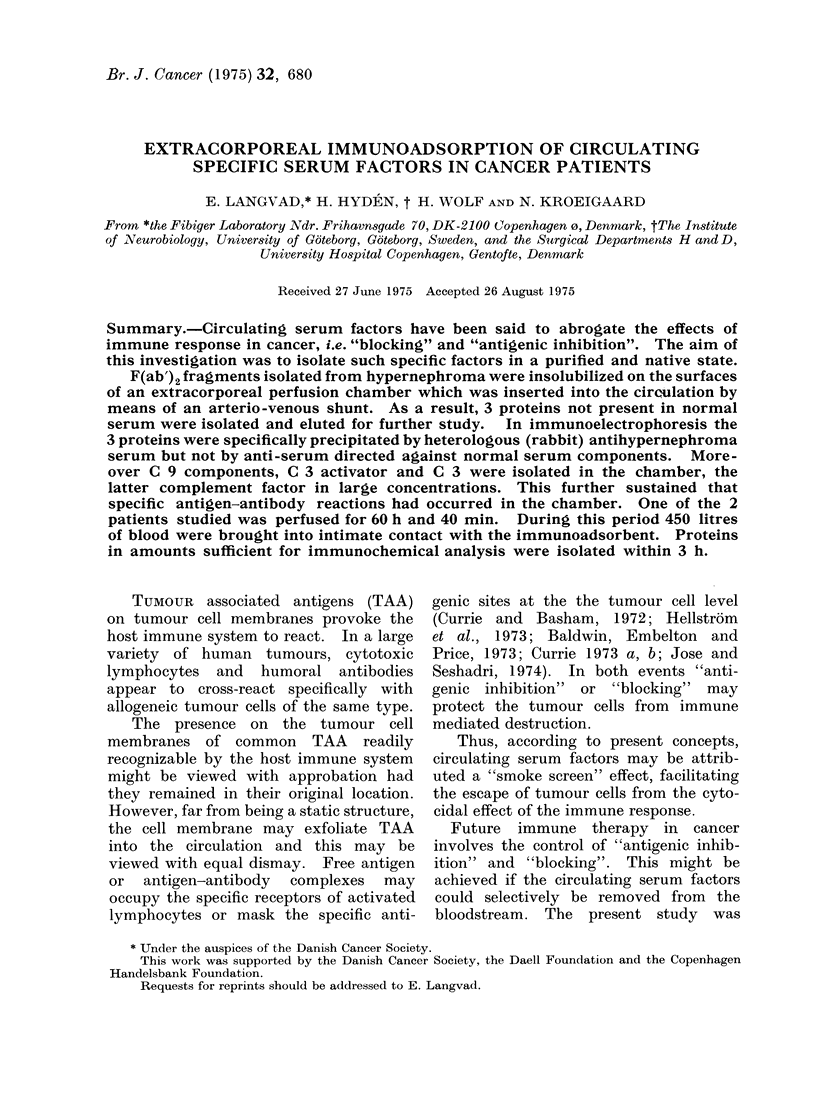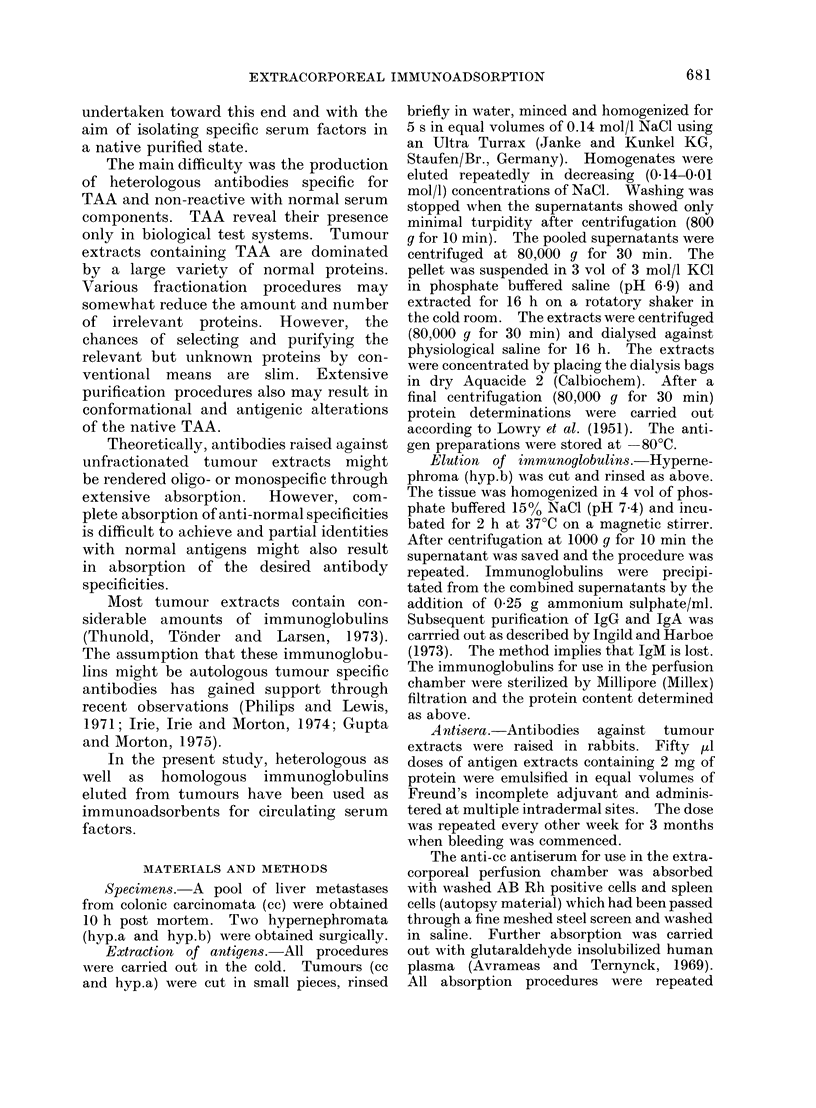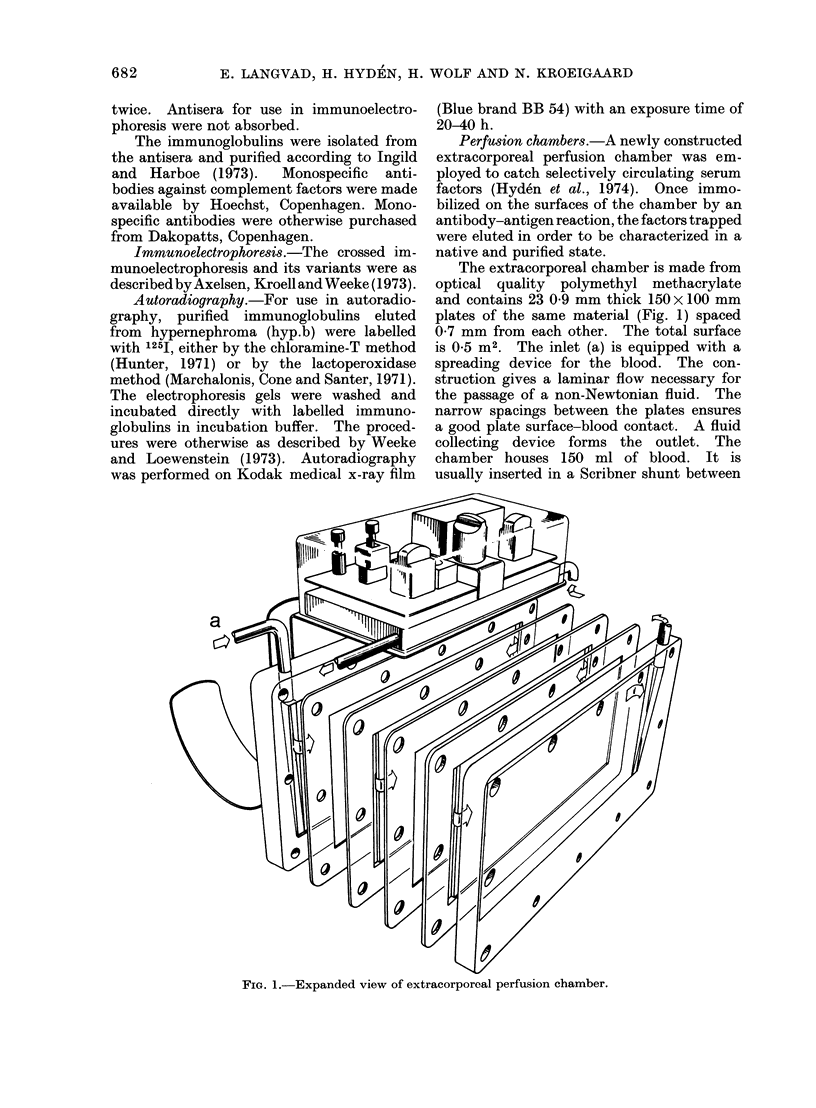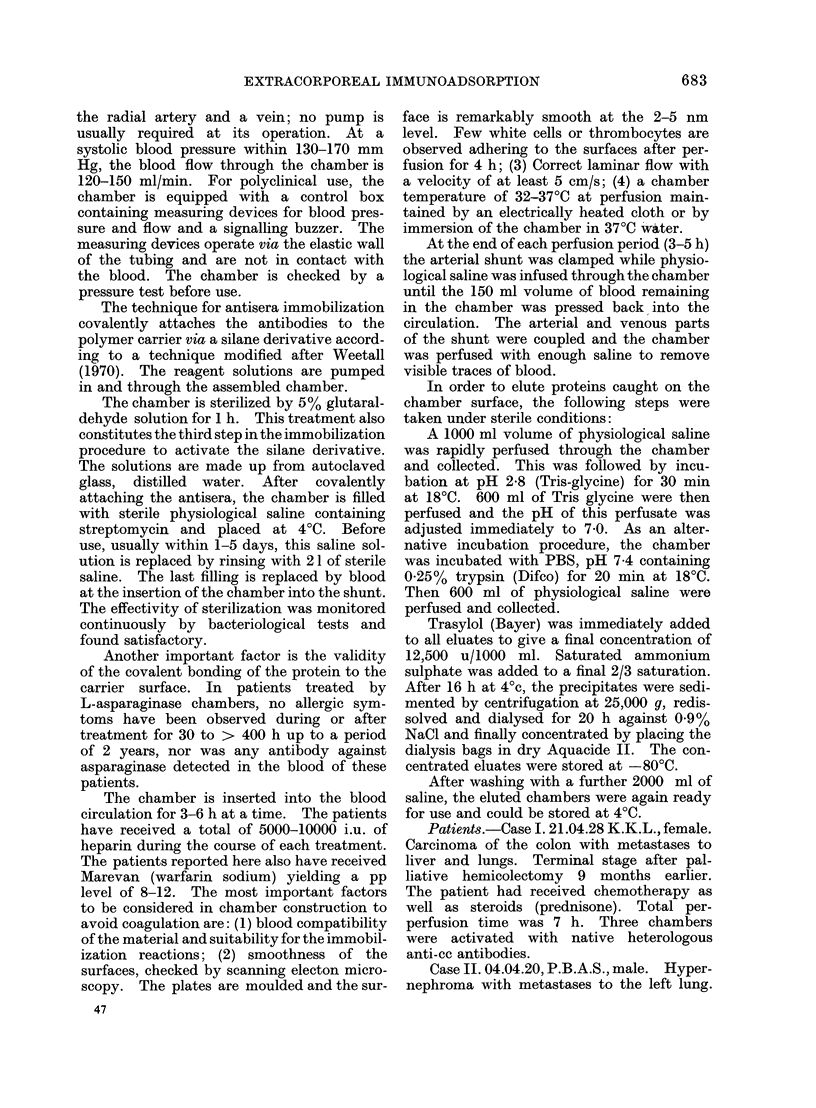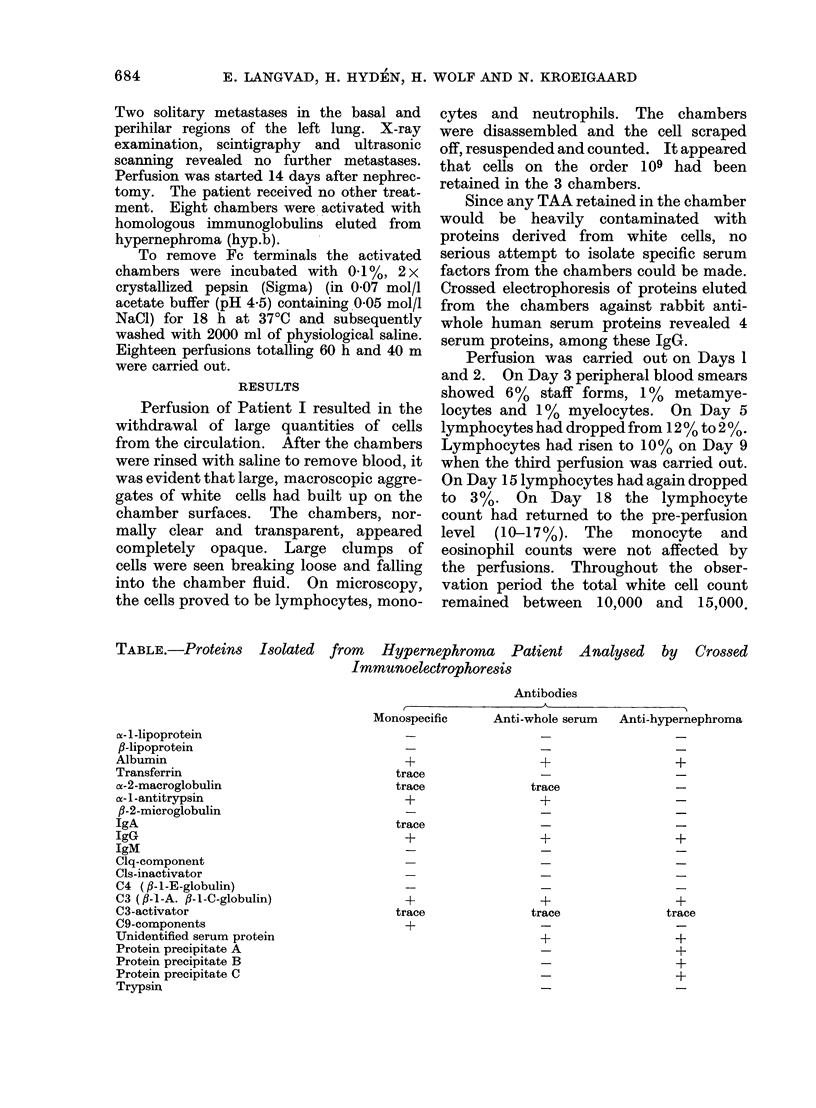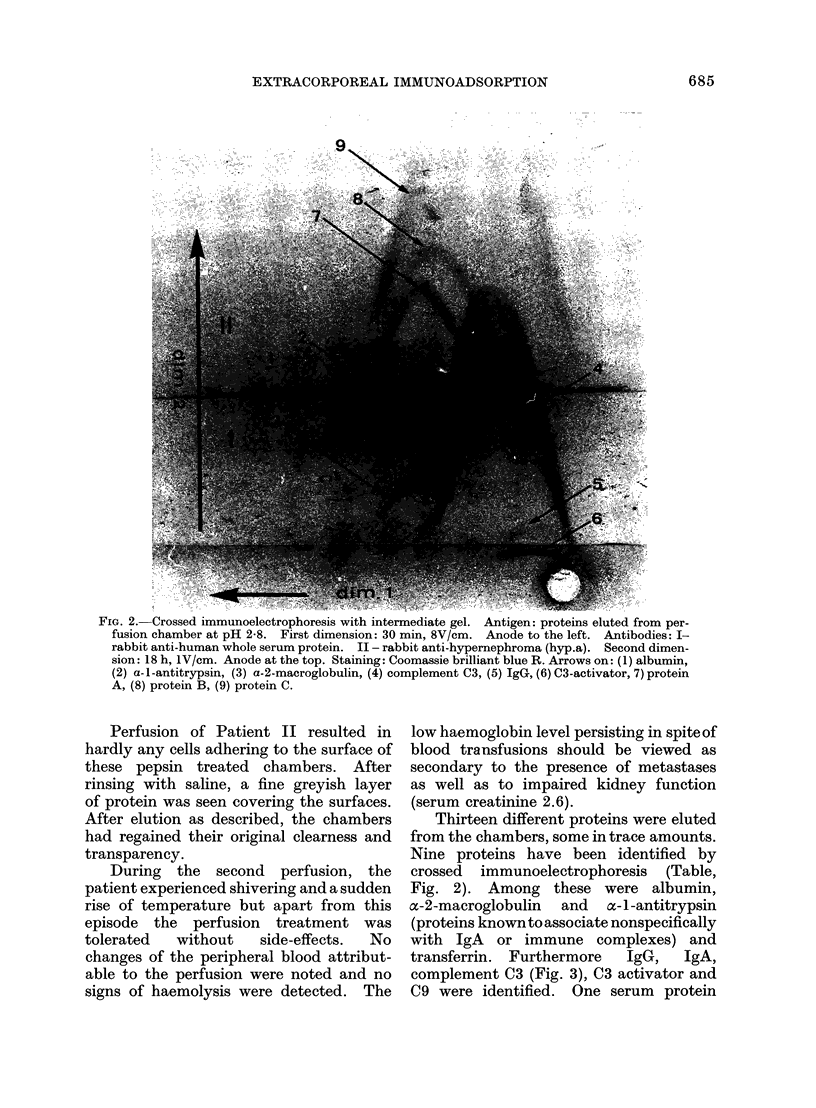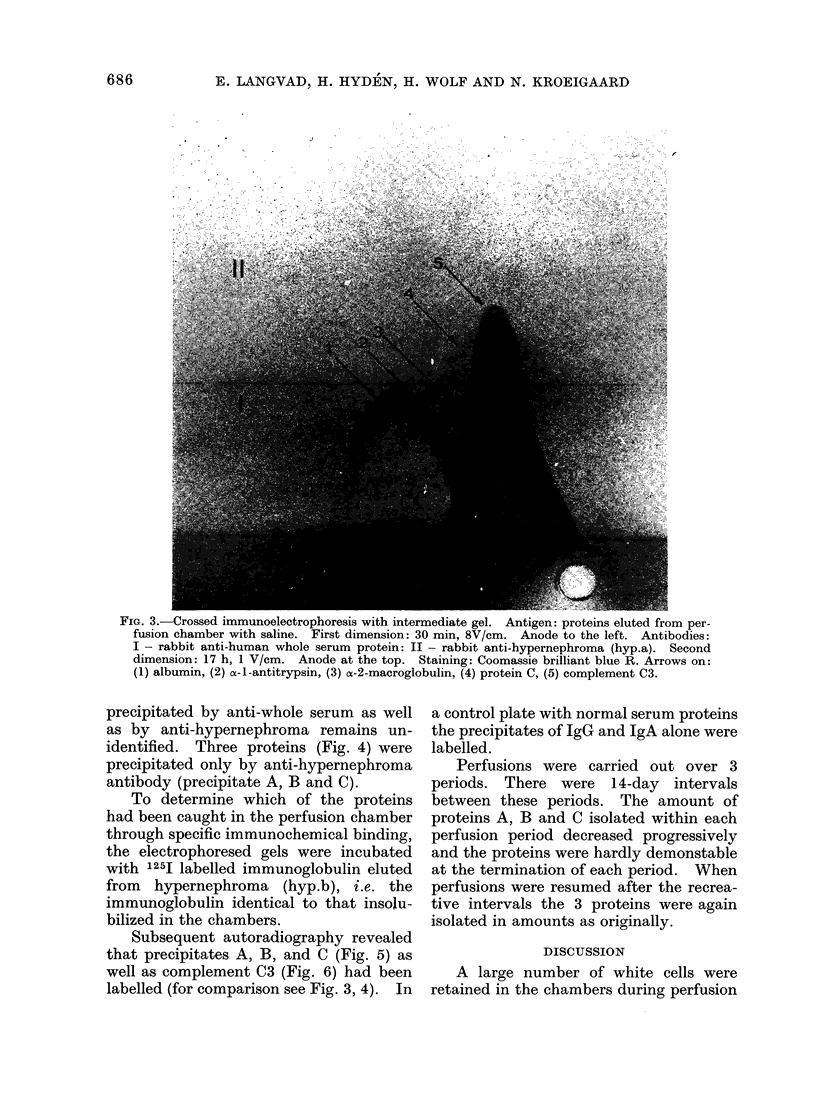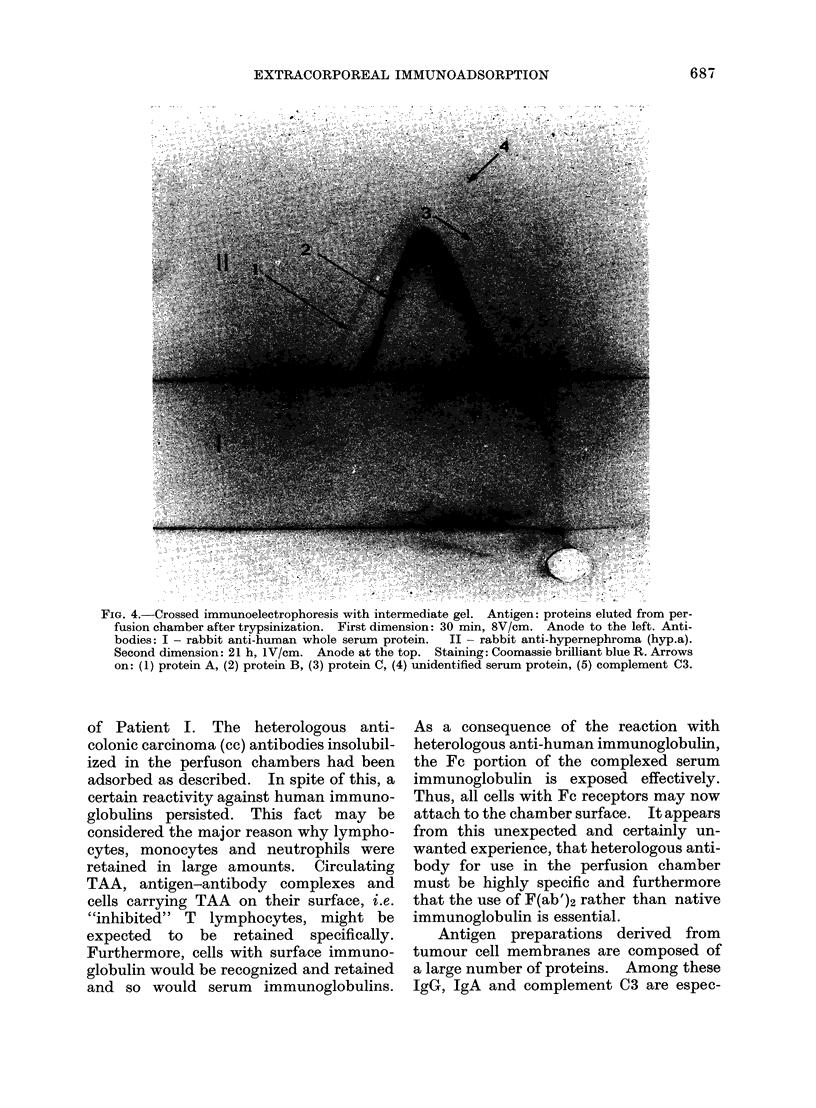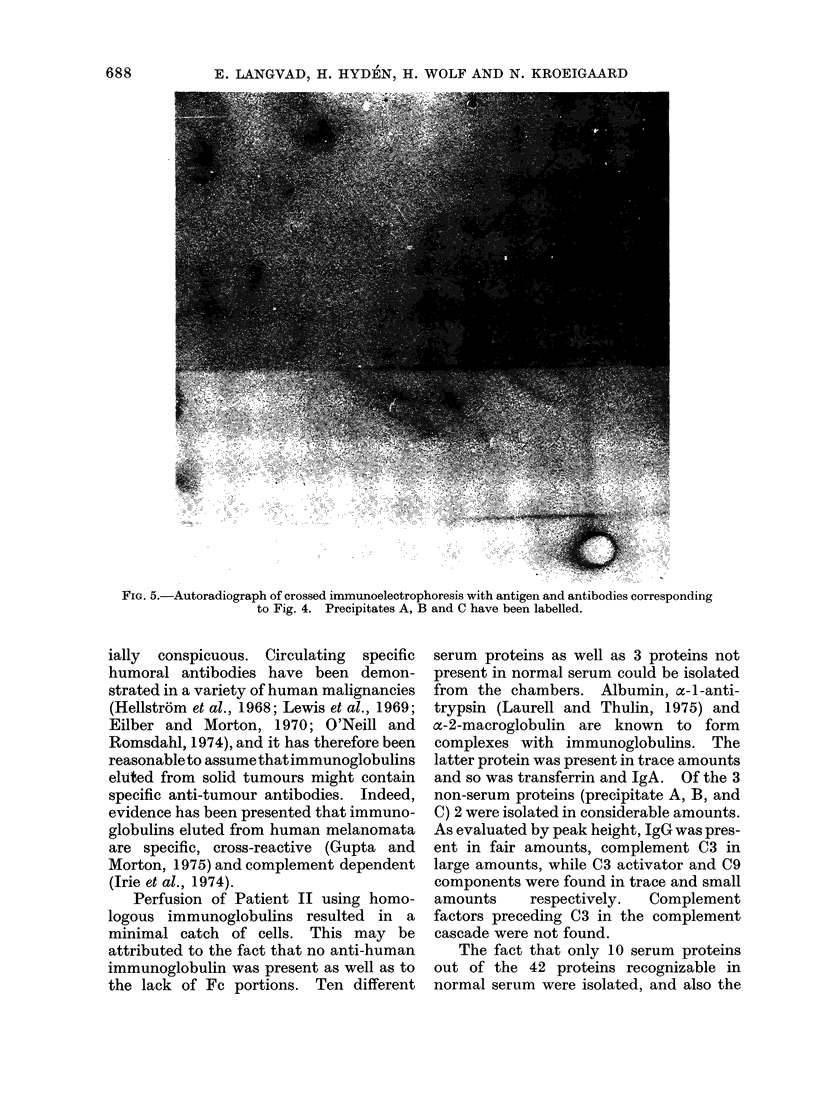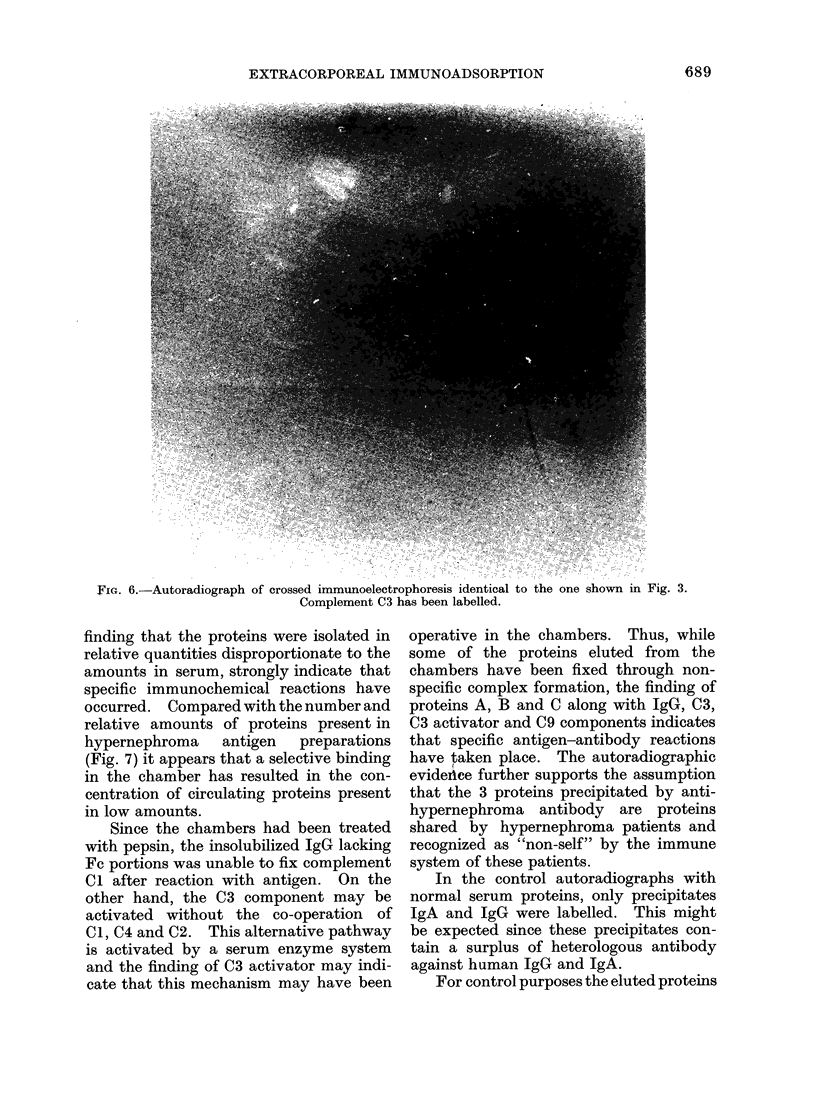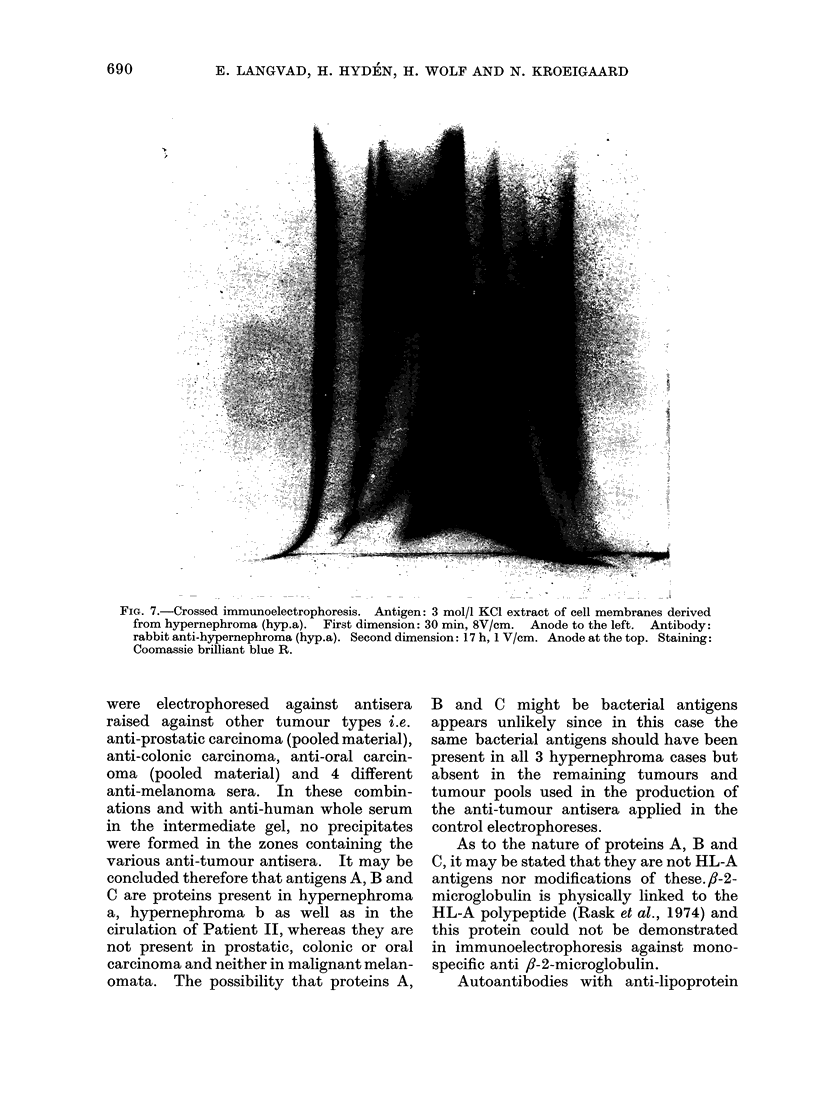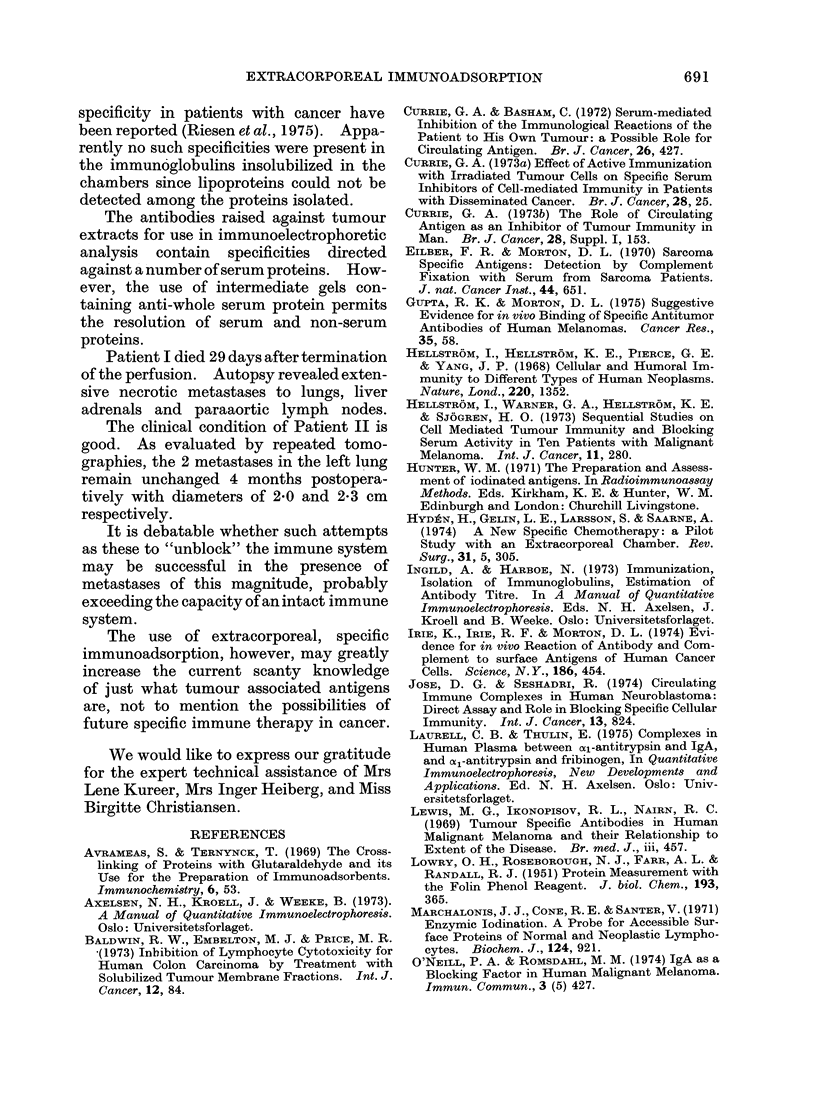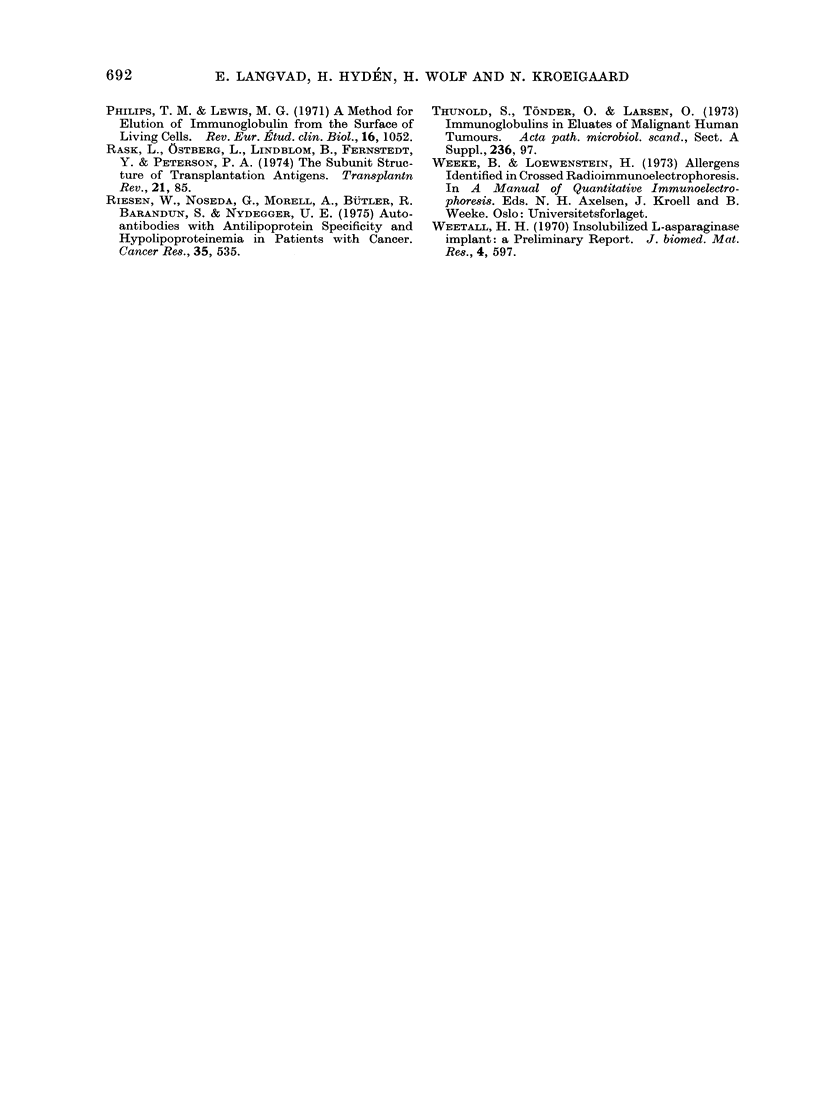# Extracorporeal immunoadsorption of circulating specific serum factors in cancer patients.

**DOI:** 10.1038/bjc.1975.279

**Published:** 1975-12

**Authors:** E. Langvad, H. Hydén, H. Wolf, N. Kroeigaard

## Abstract

**Images:**


*Br. J. Cancer* (1975) **32**, 680

### EXTRACORPOREAL IMMUNOADSORPTION OF CIRCULATING SPECIFIC SERUM FACTORS IN CANCER PATIENTS

E. LANGVAD,* H. HYDÉN, † H. WOLF AND N. KROEIGAARD

*From *the Fibiger Laboratory Ndr. Frihavnsgade 70, DK-2100 Copenhagen ø, Denmark, †The Institute of Neurobiology, University of Göteborg, Göteborg, Sweden, and the Surgical Departments H and D, University Hospital Copenhagen, Gentofte, Denmark*

Received 27 June 1975 Accepted 26 August 1975

**Summary.—Circulating serum factors have been said to abrogate the effects of immune response in cancer, *i.e.* "blocking" and "antigenic inhibition". The aim of this investigation was to isolate such specific factors in a purified and native state.**

**$F(ab')_2$ fragments isolated from hypernephroma were insolubilized on the surfaces of an extracorporeal perfusion chamber which was inserted into the circulation by means of an arterio-venous shunt. As a result, 3 proteins not present in normal serum were isolated and eluted for further study. In immunoelectrophoresis the 3 proteins were specifically precipitated by heterologous (rabbit) antihypernephroma serum but not by anti-serum directed against normal serum components. Moreover C 9 components, C 3 activator and C 3 were isolated in the chamber, the latter complement factor in large concentrations. This further sustained that specific antigen–antibody reactions had occurred in the chamber. One of the 2 patients studied was perfused for 60 h and 40 min. During this period 450 litres of blood were brought into intimate contact with the immunoadsorbent. Proteins in amounts sufficient for immunochemical analysis were isolated within 3 h.**

TUMOUR associated antigens (TAA) on tumour cell membranes provoke the host immune system to react. In a large variety of human tumours, cytotoxic lymphocytes and humoral antibodies appear to cross-react specifically with allogeneic tumour cells of the same type.

The presence on the tumour cell membranes of common TAA readily recognizable by the host immune system might be viewed with approbation had they remained in their original location. However, far from being a static structure, the cell membrane may exfoliate TAA into the circulation and this may be viewed with equal dismay. Free antigen or antigen–antibody complexes may occupy the specific receptors of activated lymphocytes or mask the specific antigenic sites at the the tumour cell level (Currie and Basham, 1972; Hellström *et al.*, 1973; Baldwin, Embelton and Price, 1973; Currie 1973 *a*, *b*; Jose and Seshadri, 1974). In both events "antigenic inhibition" or "blocking" may protect the tumour cells from immune mediated destruction.

Thus, according to present concepts, circulating serum factors may be attributed a "smoke screen" effect, facilitating the escape of tumour cells from the cytocidal effect of the immune response.

Future immune therapy in cancer involves the control of "antigenic inhibition" and "blocking". This might be achieved if the circulating serum factors could selectively be removed from the bloodstream. The present study was

* Under the auspices of the Danish Cancer Society.
This work was supported by the Danish Cancer Society, the Daell Foundation and the Copenhagen Handelsbank Foundation.
Requests for reprints should be addressed to E. Langvad.

undertaken toward this end and with the aim of isolating specific serum factors in a native purified state.

The main difficulty was the production of heterologous antibodies specific for TAA and non-reactive with normal serum components. TAA reveal their presence only in biological test systems. Tumour extracts containing TAA are dominated by a large variety of normal proteins. Various fractionation procedures may somewhat reduce the amount and number of irrelevant proteins. However, the chances of selecting and purifying the relevant but unknown proteins by conventional means are slim. Extensive purification procedures also may result in conformational and antigenic alterations of the native TAA.

Theoretically, antibodies raised against unfractionated tumour extracts might be rendered oligo- or monospecific through extensive absorption. However, complete absorption of anti-normal specificities is difficult to achieve and partial identities with normal antigens might also result in absorption of the desired antibody specificities.

Most tumour extracts contain considerable amounts of immunoglobulins (Thunold, Tönder and Larsen, 1973). The assumption that these immunoglobulins might be autologous tumour specific antibodies has gained support through recent observations (Philips and Lewis, 1971; Irie, Irie and Morton, 1974; Gupta and Morton, 1975).

In the present study, heterologous as well as homologous immunoglobulins eluted from tumours have been used as immunoadsorbents for circulating serum factors.

### MATERIALS AND METHODS

*Specimens.*—A pool of liver metastases from colonic carcinomata (cc) were obtained 10 h post mortem. Two hypernephromata (hyp.a and hyp.b) were obtained surgically.

*Extraction of antigens.*—All procedures were carried out in the cold. Tumours (cc and hyp.a) were cut in small pieces, rinsed briefly in water, minced and homogenized for 5 s in equal volumes of 0.14 mol/l NaCl using an Ultra Turrax (Janke and Kunkel KG, Staufen/Br., Germany). Homogenates were eluted repeatedly in decreasing (0·14–0·01 mol/l) concentrations of NaCl. Washing was stopped when the supernatants showed only minimal turpidity after centrifugation (800 *g* for 10 min). The pooled supernatants were centrifuged at 80,000 *g* for 30 min. The pellet was suspended in 3 vol of 3 mol/l KCl in phosphate buffered saline (pH 6·9) and extracted for 16 h on a rotatory shaker in the cold room. The extracts were centrifuged (80,000 *g* for 30 min) and dialysed against physiological saline for 16 h. The extracts were concentrated by placing the dialysis bags in dry Aquacide 2 (Calbiochem). After a final centrifugation (80,000 *g* for 30 min) protein determinations were carried out according to Lowry *et al.* (1951). The antigen preparations were stored at −80°C.

*Elution of immunoglobulins.*—Hypernephroma (hyp.b) was cut and rinsed as above. The tissue was homogenized in 4 vol of phosphate buffered 15% NaCl (pH 7·4) and incubated for 2 h at 37°C on a magnetic stirrer. After centrifugation at 1000 *g* for 10 min the supernatant was saved and the procedure was repeated. Immunoglobulins were precipitated from the combined supernatants by the addition of 0·25 g ammonium sulphate/ml. Subsequent purification of IgG and IgA was carrried out as described by Ingild and Harboe (1973). The method implies that IgM is lost. The immunoglobulins for use in the perfusion chamber were sterilized by Millipore (Millex) filtration and the protein content determined as above.

*Antisera.*—Antibodies against tumour extracts were raised in rabbits. Fifty μl doses of antigen extracts containing 2 mg of protein were emulsified in equal volumes of Freund's incomplete adjuvant and administered at multiple intradermal sites. The dose was repeated every other week for 3 months when bleeding was commenced.

The anti-cc antiserum for use in the extracorporeal perfusion chamber was absorbed with washed AB Rh positive cells and spleen cells (autopsy material) which had been passed through a fine meshed steel screen and washed in saline. Further absorption was carried out with glutaraldehyde insolubilized human plasma (Avrameas and Ternynck, 1969). All absorption procedures were repeated twice. Antisera for use in immunoelectrophoresis were not absorbed.

The immunoglobulins were isolated from the antisera and purified according to Ingild and Harboe (1973). Monospecific antibodies against complement factors were made available by Hoechst, Copenhagen. Monospecific antibodies were otherwise purchased from Dakopatts, Copenhagen.

*Immunoelectrophoresis.*—The crossed immunoelectrophoresis and its variants were as described by Axelsen, Kroell and Weeke (1973).

*Autoradiography.*—For use in autoradiography, purified immunoglobulins eluted from hypernephroma (hyp.b) were labelled with $^{125}$I, either by the chloramine-T method (Hunter, 1971) or by the lactoperoxidase method (Marchalonis, Cone and Santer, 1971). The electrophoresis gels were washed and incubated directly with labelled immunoglobulins in incubation buffer. The procedures were otherwise as described by Weeke and Loewenstein (1973). Autoradiography was performed on Kodak medical x-ray film (Blue brand BB 54) with an exposure time of 20–40 h.

*Perfusion chambers.*—A newly constructed extracorporeal perfusion chamber was employed to catch selectively circulating serum factors (Hydén *et al.*, 1974). Once immobilized on the surfaces of the chamber by an antibody–antigen reaction, the factors trapped were eluted in order to be characterized in a native and purified state.

The extracorporeal chamber is made from optical quality polymethyl methacrylate and contains 23 0·9 mm thick 150 × 100 mm plates of the same material (Fig. 1) spaced 0·7 mm from each other. The total surface is 0·5 m². The inlet (a) is equipped with a spreading device for the blood. The construction gives a laminar flow necessary for the passage of a non-Newtonian fluid. The narrow spacings between the plates ensures a good plate surface–blood contact. A fluid collecting device forms the outlet. The chamber houses 150 ml of blood. It is usually inserted in a Scribner shunt between

FIG. 1.—Expanded view of extracorporeal perfusion chamber.

the radial artery and a vein; no pump is usually required at its operation. At a systolic blood pressure within 130–170 mm Hg, the blood flow through the chamber is 120–150 ml/min. For polyclinical use, the chamber is equipped with a control box containing measuring devices for blood pressure and flow and a signalling buzzer. The measuring devices operate *via* the elastic wall of the tubing and are not in contact with the blood. The chamber is checked by a pressure test before use.

The technique for antisera immobilization covalently attaches the antibodies to the polymer carrier *via* a silane derivative according to a technique modified after Weetall (1970). The reagent solutions are pumped in and through the assembled chamber.

The chamber is sterilized by 5% glutaraldehyde solution for 1 h. This treatment also constitutes the third step in the immobilization procedure to activate the silane derivative. The solutions are made up from autoclaved glass, distilled water. After covalently attaching the antisera, the chamber is filled with sterile physiological saline containing streptomycin and placed at 4°C. Before use, usually within 1–5 days, this saline solution is replaced by rinsing with 2 l of sterile saline. The last filling is replaced by blood at the insertion of the chamber into the shunt. The effectivity of sterilization was monitored continuously by bacteriological tests and found satisfactory.

Another important factor is the validity of the covalent bonding of the protein to the carrier surface. In patients treated by L-asparaginase chambers, no allergic symtoms have been observed during or after treatment for 30 to $>$ 400 h up to a period of 2 years, nor was any antibody against asparaginase detected in the blood of these patients.

The chamber is inserted into the blood circulation for 3–6 h at a time. The patients have received a total of 5000–10000 i.u. of heparin during the course of each treatment. The patients reported here also have received Marevan (warfarin sodium) yielding a pp level of 8–12. The most important factors to be considered in chamber construction to avoid coagulation are: (1) blood compatibility of the material and suitability for the immobilization reactions; (2) smoothness of the surfaces, checked by scanning electon microscopy. The plates are moulded and the surface is remarkably smooth at the 2–5 nm level. Few white cells or thrombocytes are observed adhering to the surfaces after perfusion for 4 h; (3) Correct laminar flow with a velocity of at least 5 cm/s; (4) a chamber temperature of 32–37°C at perfusion maintained by an electrically heated cloth or by immersion of the chamber in 37°C water.

At the end of each perfusion period (3–5 h) the arterial shunt was clamped while physiological saline was infused through the chamber until the 150 ml volume of blood remaining in the chamber was pressed back into the circulation. The arterial and venous parts of the shunt were coupled and the chamber was perfused with enough saline to remove visible traces of blood.

In order to elute proteins caught on the chamber surface, the following steps were taken under sterile conditions:

A 1000 ml volume of physiological saline was rapidly perfused through the chamber and collected. This was followed by incubation at pH 2·8 (Tris-glycine) for 30 min at 18°C. 600 ml of Tris glycine were then perfused and the pH of this perfusate was adjusted immediately to 7·0. As an alternative incubation procedure, the chamber was incubated with PBS, pH 7·4 containing 0·25% trypsin (Difco) for 20 min at 18°C. Then 600 ml of physiological saline were perfused and collected.

Trasylol (Bayer) was immediately added to all eluates to give a final concentration of 12,500 u/1000 ml. Saturated ammonium sulphate was added to a final 2/3 saturation. After 16 h at 4°c, the precipitates were sedimented by centrifugation at 25,000 *g*, redissolved and dialysed for 20 h against 0·9% NaCl and finally concentrated by placing the dialysis bags in dry Aquacide II. The concentrated eluates were stored at −80°C.

After washing with a further 2000 ml of saline, the eluted chambers were again ready for use and could be stored at 4°C.

*Patients.*—Case I. 21.04.28 K.K.L., female. Carcinoma of the colon with metastases to liver and lungs. Terminal stage after palliative hemicolectomy 9 months earlier. The patient had received chemotherapy as well as steroids (prednisone). Total perperfusion time was 7 h. Three chambers were activated with native heterologous anti-cc antibodies.

Case II. 04.04.20, P.B.A.S., male. Hypernephroma with metastases to the left lung.

Two solitary metastases in the basal and perihilar regions of the left lung. X-ray examination, scintigraphy and ultrasonic scanning revealed no further metastases. Perfusion was started 14 days after nephrectomy. The patient received no other treatment. Eight chambers were activated with homologous immunoglobulins eluted from hypernephroma (hyp.b).

To remove Fc terminals the activated chambers were incubated with 0·1%, 2× crystallized pepsin (Sigma) (in 0·07 mol/l acetate buffer (pH 4·5) containing 0·05 mol/l NaCl) for 18 h at 37°C and subsequently washed with 2000 ml of physiological saline. Eighteen perfusions totalling 60 h and 40 m were carried out.

### RESULTS

Perfusion of Patient I resulted in the withdrawal of large quantities of cells from the circulation. After the chambers were rinsed with saline to remove blood, it was evident that large, macroscopic aggregates of white cells had built up on the chamber surfaces. The chambers, normally clear and transparent, appeared completely opaque. Large clumps of cells were seen breaking loose and falling into the chamber fluid. On microscopy, the cells proved to be lymphocytes, monocytes and neutrophils. The chambers were disassembled and the cell scraped off, resuspended and counted. It appeared that cells on the order $10^9$ had been retained in the 3 chambers.

Since any TAA retained in the chamber would be heavily contaminated with proteins derived from white cells, no serious attempt to isolate specific serum factors from the chambers could be made. Crossed electrophoresis of proteins eluted from the chambers against rabbit anti-whole human serum proteins revealed 4 serum proteins, among these IgG.

Perfusion was carried out on Days 1 and 2. On Day 3 peripheral blood smears showed 6% staff forms, 1% metamyelocytes and 1% myelocytes. On Day 5 lymphocytes had dropped from 12% to 2%. Lymphocytes had risen to 10% on Day 9 when the third perfusion was carried out. On Day 15 lymphocytes had again dropped to 3%. On Day 18 the lymphocyte count had returned to the pre-perfusion level (10–17%). The monocyte and eosinophil counts were not affected by the perfusions. Throughout the observation period the total white cell count remained between 10,000 and 15,000.

TABLE.—*Proteins Isolated from Hypernephroma Patient Analysed by Crossed Immunoelectrophoresis*

| | Antibodies | | |
|---|---|---|---|
| | Monospecific | Anti-whole serum | Anti-hypernephroma |
| α-1-lipoprotein | — | — | — |
| β-lipoprotein | — | — | — |
| Albumin | + | + | + |
| Transferrin | trace | — | — |
| α-2-macroglobulin | trace | trace | — |
| α-1-antitrypsin | + | + | — |
| β-2-microglobulin | — | — | — |
| IgA | trace | — | — |
| IgG | + | + | + |
| IgM | — | — | — |
| Clq-component | — | — | — |
| Cls-inactivator | — | — | — |
| C4 (β-1-E-globulin) | — | — | — |
| C3 (β-1-A. β-1-C-globulin) | + | + | + |
| C3-activator | trace | trace | trace |
| C9-components | + | — | — |
| Unidentified serum protein | | + | + |
| Protein precipitate A | | — | + |
| Protein precipitate B | | — | + |
| Protein precipitate C | | — | + |
| Trypsin | | — | — |

FIG. 2.—Crossed immunoelectrophoresis with intermediate gel. Antigen: proteins eluted from perfusion chamber at pH 2·8. First dimension: 30 min, 8V/cm. Anode to the left. Antibodies: I–rabbit anti-human whole serum protein. II – rabbit anti-hypernephroma (hyp.a). Second dimension: 18 h, 1V/cm. Anode at the top. Staining: Coomassie brilliant blue R. Arrows on: (1) albumin, (2) α-1-antitrypsin, (3) α-2-macroglobulin, (4) complement C3, (5) IgG, (6) C3-activator, 7) protein A, (8) protein B, (9) protein C.

Perfusion of Patient II resulted in hardly any cells adhering to the surface of these pepsin treated chambers. After rinsing with saline, a fine greyish layer of protein was seen covering the surfaces. After elution as described, the chambers had regained their original clearness and transparency.

During the second perfusion, the patient experienced shivering and a sudden rise of temperature but apart from this episode the perfusion treatment was tolerated without side-effects. No changes of the peripheral blood attributable to the perfusion were noted and no signs of haemolysis were detected. The low haemoglobin level persisting in spite of blood transfusions should be viewed as secondary to the presence of metastases as well as to impaired kidney function (serum creatinine 2.6).

Thirteen different proteins were eluted from the chambers, some in trace amounts. Nine proteins have been identified by crossed immunoelectrophoresis (Table, Fig. 2). Among these were albumin, α-2-macroglobulin and α-1-antitrypsin (proteins known to associate nonspecifically with IgA or immune complexes) and transferrin. Furthermore IgG, IgA, complement C3 (Fig. 3), C3 activator and C9 were identified. One serum protein

FIG. 3.—Crossed immunoelectrophoresis with intermediate gel. Antigen: proteins eluted from perfusion chamber with saline. First dimension: 30 min, 8V/cm. Anode to the left. Antibodies: I – rabbit anti-human whole serum protein: II – rabbit anti-hypernephroma (hyp.a). Second dimension: 17 h, 1 V/cm. Anode at the top. Staining: Coomassie brilliant blue R. Arrows on: (1) albumin, (2) α-1-antitrypsin, (3) α-2-macroglobulin, (4) protein C, (5) complement C3.

precipitated by anti-whole serum as well as by anti-hypernephroma remains unidentified. Three proteins (Fig. 4) were precipitated only by anti-hypernephroma antibody (precipitate A, B and C).

To determine which of the proteins had been caught in the perfusion chamber through specific immunochemical binding, the electrophoresed gels were incubated with $^{125}I$ labelled immunoglobulin eluted from hypernephroma (hyp.b), *i.e.* the immunoglobulin identical to that insolubilized in the chambers.

Subsequent autoradiography revealed that precipitates A, B, and C (Fig. 5) as well as complement C3 (Fig. 6) had been labelled (for comparison see Fig. 3, 4). In a control plate with normal serum proteins the precipitates of IgG and IgA alone were labelled.

Perfusions were carried out over 3 periods. There were 14-day intervals between these periods. The amount of proteins A, B and C isolated within each perfusion period decreased progressively and the proteins were hardly demonstable at the termination of each period. When perfusions were resumed after the recreative intervals the 3 proteins were again isolated in amounts as originally.

### DISCUSSION

A large number of white cells were retained in the chambers during perfusion

FIG. 4.—Crossed immunoelectrophoresis with intermediate gel. Antigen: proteins eluted from perfusion chamber after trypsinization. First dimension: 30 min, 8V/cm. Anode to the left. Antibodies: I – rabbit anti-human whole serum protein. II – rabbit anti-hypernephroma (hyp.a). Second dimension: 21 h, 1V/cm. Anode at the top. Staining: Coomassie brilliant blue R. Arrows on: (1) protein A, (2) protein B, (3) protein C, (4) unidentified serum protein, (5) complement C3.

of Patient I. The heterologous anti-colonic carcinoma (cc) antibodies insolubilized in the perfuson chambers had been adsorbed as described. In spite of this, a certain reactivity against human immunoglobulins persisted. This fact may be considered the major reason why lymphocytes, monocytes and neutrophils were retained in large amounts. Circulating TAA, antigen–antibody complexes and cells carrying TAA on their surface, *i.e.* "inhibited" T lymphocytes, might be expected to be retained specifically. Furthermore, cells with surface immunoglobulin would be recognized and retained and so would serum immunoglobulins. As a consequence of the reaction with heterologous anti-human immunoglobulin, the Fc portion of the complexed serum immunoglobulin is exposed effectively. Thus, all cells with Fc receptors may now attach to the chamber surface. It appears from this unexpected and certainly unwanted experience, that heterologous antibody for use in the perfusion chamber must be highly specific and furthermore that the use of $F(ab')_2$ rather than native immunoglobulin is essential.

Antigen preparations derived from tumour cell membranes are composed of a large number of proteins. Among these IgG, IgA and complement C3 are espec-

FIG. 5.—Autoradiograph of crossed immunoelectrophoresis with antigen and antibodies corresponding to Fig. 4. Precipitates A, B and C have been labelled.

ially conspicuous. Circulating specific humoral antibodies have been demonstrated in a variety of human malignancies (Hellström *et al.*, 1968; Lewis *et al.*, 1969; Eilber and Morton, 1970; O'Neill and Romsdahl, 1974), and it has therefore been reasonable to assume that immunoglobulins eluted from solid tumours might contain specific anti-tumour antibodies. Indeed, evidence has been presented that immunoglobulins eluted from human melanomata are specific, cross-reactive (Gupta and Morton, 1975) and complement dependent (Irie *et al.*, 1974).

Perfusion of Patient II using homologous immunoglobulins resulted in a minimal catch of cells. This may be attributed to the fact that no anti-human immunoglobulin was present as well as to the lack of Fc portions. Ten different serum proteins as well as 3 proteins not present in normal serum could be isolated from the chambers. Albumin, $\alpha$-1-antitrypsin (Laurell and Thulin, 1975) and $\alpha$-2-macroglobulin are known to form complexes with immunoglobulins. The latter protein was present in trace amounts and so was transferrin and IgA. Of the 3 non-serum proteins (precipitate A, B, and C) 2 were isolated in considerable amounts. As evaluated by peak height, IgG was present in fair amounts, complement C3 in large amounts, while C3 activator and C9 components were found in trace and small amounts respectively. Complement factors preceding C3 in the complement cascade were not found.

The fact that only 10 serum proteins out of the 42 proteins recognizable in normal serum were isolated, and also the

Fig. 6.—Autoradiograph of crossed immunoelectrophoresis identical to the one shown in Fig. 3. Complement C3 has been labelled.

finding that the proteins were isolated in relative quantities disproportionate to the amounts in serum, strongly indicate that specific immunochemical reactions have occurred. Compared with the number and relative amounts of proteins present in hypernephroma antigen preparations (Fig. 7) it appears that a selective binding in the chamber has resulted in the concentration of circulating proteins present in low amounts.

Since the chambers had been treated with pepsin, the insolubilized IgG lacking Fc portions was unable to fix complement C1 after reaction with antigen. On the other hand, the C3 component may be activated without the co-operation of C1, C4 and C2. This alternative pathway is activated by a serum enzyme system and the finding of C3 activator may indicate that this mechanism may have been operative in the chambers. Thus, while some of the proteins eluted from the chambers have been fixed through non-specific complex formation, the finding of proteins A, B and C along with IgG, C3, C3 activator and C9 components indicates that specific antigen–antibody reactions have taken place. The autoradiographic evidence further supports the assumption that the 3 proteins precipitated by anti-hypernephroma antibody are proteins shared by hypernephroma patients and recognized as "non-self" by the immune system of these patients.

In the control autoradiographs with normal serum proteins, only precipitates IgA and IgG were labelled. This might be expected since these precipitates contain a surplus of heterologous antibody against human IgG and IgA.

For control purposes the eluted proteins

FIG. 7.—Crossed immunoelectrophoresis. Antigen: 3 mol/l KCl extract of cell membranes derived from hypernephroma (hyp.a). First dimension: 30 min, 8V/cm. Anode to the left. Antibody: rabbit anti-hypernephroma (hyp.a). Second dimension: 17 h, 1 V/cm. Anode at the top. Staining: Coomassie brilliant blue R.

were electrophoresed against antisera raised against other tumour types *i.e.* anti-prostatic carcinoma (pooled material), anti-colonic carcinoma, anti-oral carcinoma (pooled material) and 4 different anti-melanoma sera. In these combinations and with anti-human whole serum in the intermediate gel, no precipitates were formed in the zones containing the various anti-tumour antisera. It may be concluded therefore that antigens A, B and C are proteins present in hypernephroma a, hypernephroma b as well as in the cirulation of Patient II, whereas they are not present in prostatic, colonic or oral carcinoma and neither in malignant melanomata. The possibility that proteins A, B and C might be bacterial antigens appears unlikely since in this case the same bacterial antigens should have been present in all 3 hypernephroma cases but absent in the remaining tumours and tumour pools used in the production of the anti-tumour antisera applied in the control electrophoreses.

As to the nature of proteins A, B and C, it may be stated that they are not HL-A antigens nor modifications of these. $\beta$-2-microglobulin is physically linked to the HL-A polypeptide (Rask *et al.*, 1974) and this protein could not be demonstrated in immunoelectrophoresis against monospecific anti $\beta$-2-microglobulin.

Autoantibodies with anti-lipoprotein specificity in patients with cancer have been reported (Riesen *et al.*, 1975). Apparently no such specificities were present in the immunoglobulins insolubilized in the chambers since lipoproteins could not be detected among the proteins isolated.

The antibodies raised against tumour extracts for use in immunoelectrophoretic analysis contain specificities directed against a number of serum proteins. However, the use of intermediate gels containing anti-whole serum protein permits the resolution of serum and non-serum proteins.

Patient I died 29 days after termination of the perfusion. Autopsy revealed extensive necrotic metastases to lungs, liver adrenals and paraaortic lymph nodes.

The clinical condition of Patient II is good. As evaluated by repeated tomographies, the 2 metastases in the left lung remain unchanged 4 months postoperatively with diameters of 2·0 and 2·3 cm respectively.

It is debatable whether such attempts as these to "unblock" the immune system may be successful in the presence of metastases of this magnitude, probably exceeding the capacity of an intact immune system.

The use of extracorporeal, specific immunoadsorption, however, may greatly increase the current scanty knowledge of just what tumour associated antigens are, not to mention the possibilities of future specific immune therapy in cancer.

We would like to express our gratitude for the expert technical assistance of Mrs Lene Kureer, Mrs Inger Heiberg, and Miss Birgitte Christiansen.

### REFERENCES

AVRAMEAS, S. & TERNYNCK, T. (1969) The Cross-linking of Proteins with Glutaraldehyde and its Use for the Preparation of Immunoadsorbents. *Immunochemistry*, **6**, 53.

AXELSEN, N. H., KROELL, J. & WEEKE, B. (1973). *A Manual of Quantitative Immunoelectrophoresis*. Oslo: Universitetsforlaget.

BALDWIN, R. W., EMBELTON, M. J. & PRICE, M. R. (1973) Inhibition of Lymphocyte Cytotoxicity for Human Colon Carcinoma by Treatment with Solubilized Tumour Membrane Fractions. *Int. J. Cancer*, **12**, 84.

CURRIE, G. A. & BASHAM, C. (1972) Serum-mediated Inhibition of the Immunological Reactions of the Patient to His Own Tumour: a Possible Role for Circulating Antigen. *Br. J. Cancer*, **26**, 427.

CURRIE, G. A. (1973*a*) Effect of Active Immunization with Irradiated Tumour Cells on Specific Serum Inhibitors of Cell-mediated Immunity in Patients with Disseminated Cancer. *Br. J. Cancer*, **28**, 25.

CURRIE, G. A. (1973*b*) The Role of Circulating Antigen as an Inhibitor of Tumour Immunity in Man. *Br. J. Cancer*, **28**, Suppl. I, 153.

EILBER, F. R. & MORTON, D. L. (1970) Sarcoma Specific Antigens: Detection by Complement Fixation with Serum from Sarcoma Patients. *J. nat. Cancer Inst.*, **44**, 651.

GUPTA, R. K. & MORTON, D. L. (1975) Suggestive Evidence for *in vivo* Binding of Specific Antitumor Antibodies of Human Melanomas. *Cancer Res.*, **35**, 58.

HELLSTRÖM, I., HELLSTRÖM, K. E., PIERCE, G. E. & YANG, J. P. (1968) Cellular and Humoral Immunity to Different Types of Human Neoplasms. *Nature, Lond.*, **220**, 1352.

HELLSTRÖM, I., WARNER, G. A., HELLSTRÖM, K. E. & SJÖGREN, H. O. (1973) Sequential Studies on Cell Mediated Tumour Immunity and Blocking Serum Activity in Ten Patients with Malignant Melanoma. *Int. J. Cancer*, **11**, 280.

HUNTER, W. M. (1971) The Preparation and Assessment of iodinated antigens. In *Radioimmunoassay Methods*. Eds. Kirkham, K. E. & Hunter, W. M. Edinburgh and London: Churchill Livingstone.

HYDÉN, H., GELIN, L. E., LARSSON, S. & SAARNE, A. (1974) A New Specific Chemotherapy: a Pilot Study with an Extracorporeal Chamber. *Rev. Surg.*, **31**, 5, 305.

INGILD, A. & HARBOE, N. (1973) Immunization, Isolation of Immunoglobulins, Estimation of Antibody Titre. In *A Manual of Quantitative Immunoelectrophoresis*. Eds. N. H. Axelsen, J. Kroell and B. Weeke. Oslo: Universitetsforlaget.

IRIE, K., IRIE, R. F. & MORTON, D. L. (1974) Evidence for *in vivo* Reaction of Antibody and Complement to surface Antigens of Human Cancer Cells. *Science, N.Y.*, **186**, 454.

JOSE, D. G. & SESHADRI, R. (1974) Circulating Immune Complexes in Human Neuroblastoma: Direct Assay and Role in Blocking Specific Cellular Immunity. *Int. J. Cancer*, **13**, 824.

LAURELL, C. B. & THULIN, E. (1975) Complexes in Human Plasma between $\alpha_1$-antitrypsin and IgA, and $\alpha_1$-antitrypsin and fribinogen, In *Quantitative Immunoelectrophoresis, New Developments and Applications*. Ed. N. H. Axelsen. Oslo: Universitetsforlaget.

LEWIS, M. G., IKONOPISOV, R. L., NAIRN, R. C. (1969) Tumour Specific Antibodies in Human Malignant Melanoma and their Relationship to Extent of the Disease. *Br. med. J.*, iii, 457.

LOWRY, O. H., ROSEBOROUGH, N. J., FARR, A. L. & RANDALL, R. J. (1951) Protein Measurement with the Folin Phenol Reagent. *J. biol. Chem.*, **193**, 365.

MARCHALONIS, J. J., CONE, R. E. & SANTER, V. (1971) Enzymic Iodination. A Probe for Accessible Surface Proteins of Normal and Neoplastic Lymphocytes. *Biochem. J.*, **124**, 921.

O'NEILL, P. A. & ROMSDAHL, M. M. (1974) IgA as a Blocking Factor in Human Malignant Melanoma. *Immun. Commun.*, **3** (5) 427.

Philips, T. M. & Lewis, M. G. (1971) A Method for Elution of Immunoglobulin from the Surface of Living Cells. *Rev. Eur. Étud. clin. Biol.*, **16**, 1052.

Rask, L., Östberg, L., Lindblom, B., Fernstedt, Y. & Peterson, P. A. (1974) The Subunit Structure of Transplantation Antigens. *Transplantn Rev.*, **21**, 85.

Riesen, W., Noseda, G., Morell, A., Bütler, R. Barandun, S. & Nydegger, U. E. (1975) Autoantibodies with Antilipoprotein Specificity and Hypolipoproteinemia in Patients with Cancer. *Cancer Res.*, **35**, 535.

Thunold, S., Tönder, O. & Larsen, O. (1973) Immunoglobulins in Eluates of Malignant Human Tumours. *Acta path. microbiol. scand.*, Sect. A Suppl., **236**, 97.

Weeke, B. & Loewenstein, H. (1973) Allergens Identified in Crossed Radioimmunoelectrophoresis. In *A Manual of Quantitative Immunoelectrophoresis*. Eds. N. H. Axelsen, J. Kroell and B. Weeke. Oslo: Universitetsforlaget.

Weetall, H. H. (1970) Insolubilized L-asparaginase implant: a Preliminary Report. *J. biomed. Mat. Res.*, **4**, 597.